# Ocular and Periocular Tattoo Adverse Effects: A Review

**DOI:** 10.3390/diagnostics14192150

**Published:** 2024-09-27

**Authors:** Kar Wai Alvin Lee, Lisa Kwin Wah Chan, Angela Wai Kay Lee, Cheuk Hung Lee, Jovian Wan, Kyu-Ho Yi

**Affiliations:** 1 EverKeen Medical Centre, Hong Kong; alvin429@yahoo.com (K.W.A.L.); drchan.everkeen@gmail.com (L.K.W.C.); andylee618@hotmail.com (C.H.L.); 2 The Skin Oracle, Hong Kong; ang3la.l33@gmail.com; 3 Asia Pacific Aesthetic Academy, Hong Kong; jovian.wan@apaa.org; 4 Division in Anatomy and Developmental Biology, Department of Oral Biology, Human Identification Research Institute, BK21 FOUR Project, Yonsei University College of Dentistry, 50-1 Yonsei-ro, Seodaemun-gu, Seoul 03722, Republic of Korea; 5 Maylin Clinic (Apgujeong), Seoul 06001, Republic of Korea

**Keywords:** ocular tattooing, periocular tattooing, complications, visual impairment, infection risks

## Abstract

Background: Ocular and periocular tattoos, involving ink application to the eyeball or surrounding skin, have gained popularity as forms of self-expression. However, this trend raises significant concerns about potential complications that can adversely affect ocular health and esthetics. Awareness of these risks is crucial for both patients and practitioners. Methods: A comprehensive literature review was conducted, focusing on studies discussing complications related to ocular and periocular tattooing. Relevant studies were identified through the MEDLINE, PubMed, and Ovid databases. The reviewed papers were evaluated based on study design, including blinding, sample size, control use, randomization, and objective endpoints, and classified according to the Oxford Center for Evidence-Based Medicine evidence hierarchy. Results: The review identified a wide range of complications, including immediate issues like bleeding, infections (conjunctivitis, endophthalmitis), and allergic reactions. Delayed reactions included granuloma formation, often requiring further treatment. The most serious risk identified was potential visual impairment due to improper technique or ink placement. Conclusions: With the growing trend in ocular and periocular tattooing, there is an urgent need for increased awareness of associated risks. It is crucial to ensure that only qualified professionals perform these procedures, emphasizing the importance of understanding ocular anatomy. Developing strict regulatory guidelines and prioritizing research on the long-term effects of these tattoos are essential for patient safety. A collaborative approach among healthcare providers, regulatory bodies, and educational institutions is needed to mitigate risks and promote best practices in cosmetic tattooing.

## 1. Introduction

Ocular and periocular tattooing, including scleral, conjunctival, corneal, and eyelid tattoos, has seen a rise in popularity globally, driven by cultural trends in body modification and cosmetic enhancements. Although specific prevalence data are limited, available studies and surveys indicate that these types of tattoos are relatively rare compared to more conventional body tattoos. Estimates suggest that ocular tattooing procedures represent less than 1% of the overall tattooing market [1]. A study reported that approximately 0.05% of individuals with body modifications have undergone scleral or conjunctival tattooing, while periocular tattoos such as those on the eyelids or eyebrows are more common, particularly in cosmetic applications like permanent makeup. However, since most available data are derived from case reports rather than large-scale studies, statistical data are limited [1].

Ocular tattoos are a unique form of body art that involves the application of pigment to the sclera or conjunctiva [1]. This form of tattooing is markedly different from traditional tattoos, which are typically applied to the skin. Ocular tattooing has gained traction in recent years, sparking both intrigue and controversy within the body art community and the medical world [2]. 

### 1.1. Types of Ocular Tattoos According to Anatomy

When discussing ocular tattoos, it is essential to differentiate between various methodologies and anatomical considerations. These tattoos can be categorized based on their placement and the structures they involve.

#### 1.1.1. Scleral Tattoos

**Figure 1:**
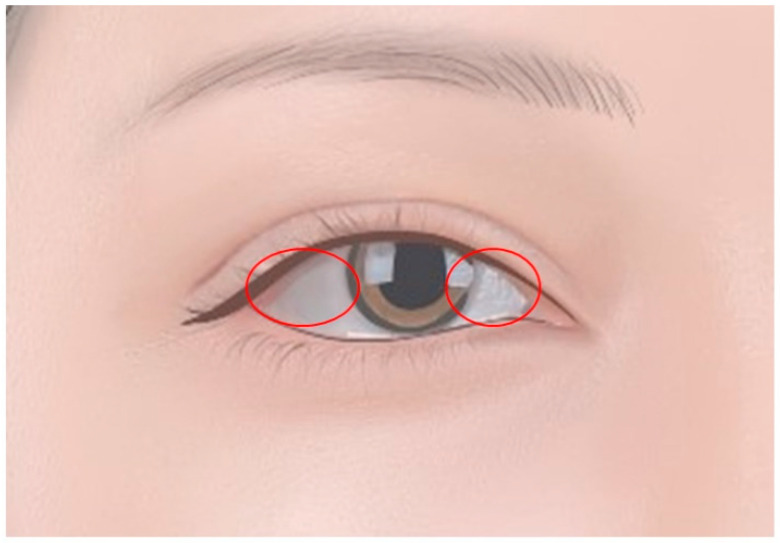
Scleral tattoos involve injecting pigment into the sclera (the white part of the eye) for cosmetic purposes, while conjunctival tattoos are less common and involve coloring the conjunctiva, the clear membrane covering the sclera and inner eyelids. The encircled areas in red indicate the locations of scleral and conjunctival tattoos, with conjunctival tattoos being more superficial and scleral tattoos located deeper.

The most common form of ocular tattooing, scleral tattoos, involve the introduction of pigment directly into the sclera (Figure 1). This process can change the eye’s appearance by covering up the natural white portion with different colors, often deep black or vivid hues. This form of tattooing is performed by ophthalmic surgeons who insert a specially formulated dye into the scleral tissue. Scleral tattoos can be particularly attractive to individuals who wish to express their identity or challenge conventional esthetics. However, they come with significant risks, including potential sight impairment, infection, and inflammation [3,4].

#### 1.1.2. Conjunctival Tattoos

Although less common, conjunctival tattoos involve coloring the conjunctiva, the thin membrane covering the sclera and inner eyelids. Unlike scleral tattoos, conjunctival tattoos are typically less invasive and may use semi-permanent dyes. The application is designed to enhance cosmetic appearance rather than changing the sclera’s color. Conjunctival tattoos provide a subtler effect compared to scleral tattoos and require a careful approach to avoid complications such as irritation or excessive tearing [5,6].

#### 1.1.3. Corneal Tattoos

**Figure 2:**
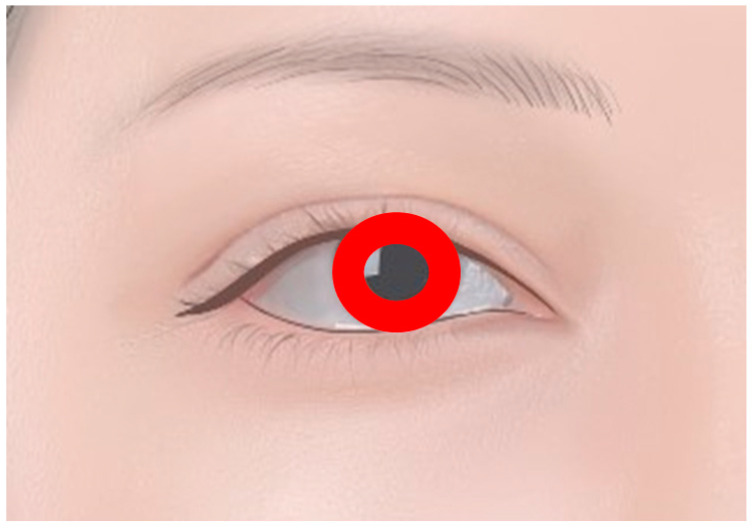
The encircled areas in red indicate the locations of corneal tattoos. A corneal tattoo is a procedure in which dye or pigment is implanted into the cornea, primarily for cosmetic or therapeutic purposes to improve the eye’s appearance or function.

In some cases, corneal tattoos are utilized to treat opacity or damage to the cornea rather than for purely esthetic reasons (Figure 2). This procedure involves implanting a pigment into the cornea to mask scarring or visual impairment. Nonetheless, this technique is highly specialized and should be distinguished from purely decorative tattoos, as it is primarily therapeutic and performed under stringent medical criteria to restore function rather than esthetics [7,8].

Periocular tattoos refer to tattoos that are applied around the eye area, specifically in the regions surrounding the eyes, including the upper eyelids, lower eyelids, and the skin adjacent to the eyes. Periocular tattoos are primarily cosmetic, aimed at enhancing the esthetic appearance of the eyes or providing certain functional benefits.

### 1.2. Periocular Tattoos

#### 1.2.1. Eyelid Tattoos

**Figure 3:**
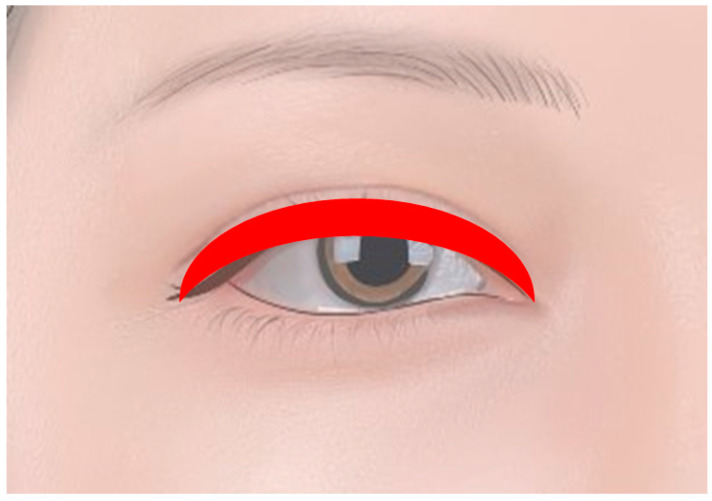
Eyelid tattoos, also known as permanent eyeliner tattoos, involve implanting pigment along the lash line of the upper and/or lower eyelids to create the appearance of defined eyeliner without the need for daily application (area colored in red).

One of the most popular forms of periocular tattoos is the permanent eyelid tattoo (eyeliner tattoos). This technique involves implanting pigment into the skin along the lash line of the upper and/or lower eyelids (Figure 3). The primary aim is to create the appearance of defined, perfectly applied eyeliner without the daily hassle of application. Eyelid tattoos can be subtle or bold, allowing for customization based on individual preferences. The procedure often requires touch-ups over time, as pigments may fade due to sun exposure and natural skin turnover [9,10].

#### 1.2.2. Eyebrow Tattoos

**Figure 4:**
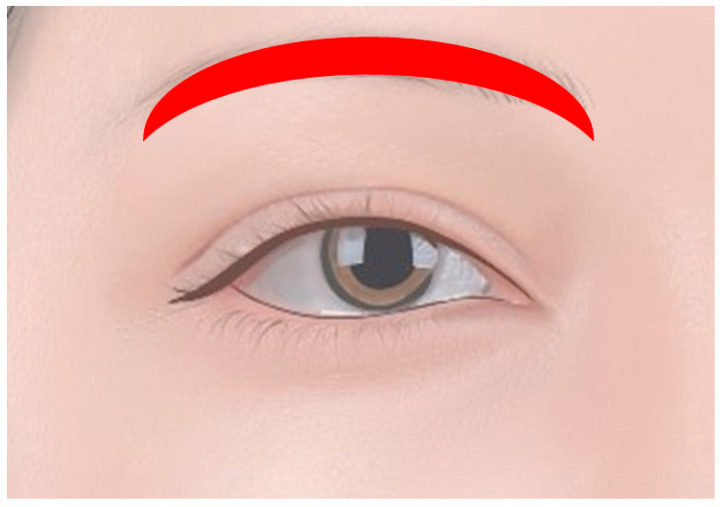
Eyebrow tattoos, including techniques like microblading, involve implanting pigment into the skin to create the appearance of fuller, well-defined eyebrows, enhancing facial esthetics and reducing the need for daily makeup (area colored in red).

While not strictly within the periocular space, eyebrow tattoos are closely associated and can significantly enhance the overall look of the eyes (Figure 4). Techniques such as microblading, where fine strokes of pigment are implanted into the skin to resemble natural hair, have become increasingly popular. Well-defined eyebrows can frame the eyes and create a more balanced facial appearance.

In the world of body art, ocular tattoos stand out as a fascinating yet controversial practice. With types vary from scleral and conjunctival tattoos to those associated with corneal adjustments, the anatomy of the eye significantly influences each tattoo’s approach and implications. While the expressive potential of these tattoos is captivating, individuals considering this form of body art must weigh esthetic desires against the inherent risks. Education and consultation with qualified professionals are paramount to ensuring safety and minimizing adverse outcomes. As this trend evolves, ongoing dialog surrounding the ethics and safety of ocular tattoos will likely continue to be a hot topic within both the art and medical communities.

## 2. Materials and Methods

**Figure 5:**
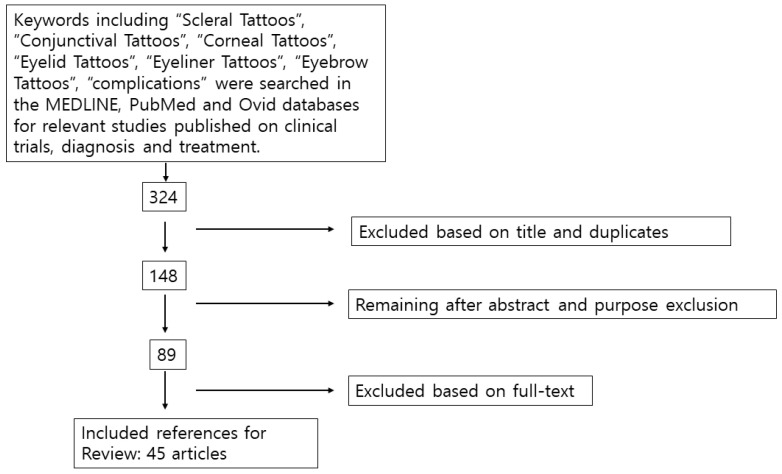
PRISMA flow diagram illustrating the selection process of relevant studies on scleral, conjunctival, corneal, eyelid, eyeliner, and eyebrow tattoos, including associated complications. Studies were identified through searches in the MEDLINE, PubMed, and Ovid databases, reviewed against clinical trial criteria, and classified according to the Oxford Center for Evidence-Based Medicine hierarchy.

The inclusion criteria for the study focused on articles related to scleral, conjunctival, corneal, eyelid, eyeliner, and eyebrow tattoos, specifically those discussing complications associated with these procedures. Relevant studies published on clinical trials, diagnosis, and treatment were selected from the MEDLINE, PubMed, and Ovid databases. Exclusion criteria involved removing duplicates and articles deemed irrelevant based on their titles. Further exclusions were made after reviewing abstracts to ensure alignment with the study’s purpose, and a final exclusion round was conducted based on full-text reviews to refine the selection to the most relevant and high-quality studies. This process narrowed the initial 324 articles down to 45 that were included in the review (Figure 5) [11]. The summary in Table 1 shows the articles included in the study.

## 3. Results

### 3.1. Scleral Tattoo Adverse Effects

Carlisle et al. [4] present a rare and significant case of an ocular complication resulting from the cosmetic practice of scleral tattooing, which involves the injection of pigment into the sclera. The authors detail the clinical case of a patient who underwent scleral tattooing and subsequently experienced an inflammatory response characterized by granuloma formation. This reaction was consistent with sarcoidosis, a condition marked by the formation of small clusters of inflammatory cells (granulomas) in various tissues, including the eyes. The patient presented with symptoms including discomfort, redness, and changes in vision, prompting further investigation.

Diagnostic procedures, including imaging and histopathological examination, confirmed the presence of a sarcoid-like granulomatous reaction localized to the scleral area where the tattoo pigment was injected. The identification of this condition is vital, as it can mimic other ocular inflammatory diseases, leading to potential misdiagnosis if not appropriately recognized. The article emphasizes the importance of considering the risks associated with scleral tattooing, particularly the potential for severe inflammatory reactions. The authors advocate for heightened awareness among both practitioners and patients regarding the complications that can arise from this cosmetic procedure. They underscore the need for proper patient education about the risks involved, as well as a thorough evaluation of any ocular symptoms following scleral tattooing.

Furthermore, the authors suggest that more stringent guidelines and regulations may be necessary to mitigate the risks associated with such cosmetic practices. As scleral tattooing becomes more popular, it is crucial to document and understand the adverse effects to inform best practices and protect patient eye health (Level IV).

Rohl et al. [3] examine the adverse effects associated with scleral tattooing using pen ink, a practice that poses significant risks to ocular health. This paper highlights two cases where patients opted for scleral tattoos for esthetic purposes, using non-medical-grade ink, ultimately leading to severe complications.

Both cases presented in the article reveal the considerable dangers of using inappropriate materials for ocular tattooing. The first patient experienced an infectious complication following the procedure, necessitating intense medical intervention. The introduction of non-sterile ink into the ocular environment raises serious concerns about infection and the potential for deleterious inflammatory responses. The second patient similarly encountered issues, reporting discomfort and inflammation, indicative of a foreign body reaction to the ink injected into the sclera.

The authors stress that existing literature reflects a growing trend towards scleral tattooing performed by untrained individuals who frequently lack an understanding of the anatomical and physiological implications of injecting foreign substances into the eye. The use of pen ink, which is not intended for medical use, compounds the risk of complications. The cases highlighted in this report illustrate that complications can range from mild irritations to severe sight-threatening situations, underscoring the unpredictability and potential severity of adverse reactions in ocular tattooing procedures. The authors advocate for heightened awareness among potential patients regarding the risks associated with scleral tattoos and emphasize the importance of using only medically verified materials and techniques. They call for better regulatory measures governing such esthetic procedures. The authors advocate for informing patients about potential complications, as the adverse effects from improperly performed ocular tattoos could lead to long-term vision problems or even permanent loss of sight (Level IV).

Haq et al. [14] explore a significant ocular complication arising from scleral tattooing, a cosmetic procedure involving the injection of pigment into the sclera. The authors document a case of delayed granulomatous anterior uveitis that occurred after an inadvertent intraocular injection of tattoo ink, highlighting the potential risks associated with this practice. In this case, report, the patient underwent scleral tattooing, which was intended solely for esthetic reasons. However, during the procedure, a portion of the tattoo ink was accidentally injected into the anterior chamber of the eye. Following this incident, the patient experienced symptoms of inflammation, including eye pain, photophobia, and decreased vision. These symptoms prompted an urgent ophthalmological examination.

The examination revealed findings consistent with anterior uveitis, specifically the iris and ciliary body. Further clinical evaluation confirmed the presence of granulomas, which are small clusters of inflammatory cells that form in response to irritation or injury. In this case, the granulomatous reaction was attributed to the presence of foreign tattoo ink within the ocular tissue, leading to an immune response that manifested as uveitis.

The authors emphasize the importance of recognizing this adverse effect as a potential complication of scleral tattooing. They note that the occurrence of delayed granulomatous inflammation following ocular exposure to tattoo ink is not well documented in the literature, making this case particularly underscored as a warning to practitioners and patients alike. The authors advocate for increased awareness and patient education about the risks associated with scleral tattooing procedures, including the possibility of inadvertent intraocular injection and the subsequent inflammatory reactions that could arise (Level IV).

Duarte et al. [15] explore the immediate complications resulting from the cosmetic procedure known as eyeball tattooing or scleral tattooing, which involves injecting pigment into the sclera. The authors present two detailed case reports illustrating the short-term complications experienced by patients who underwent this procedure. Both cases highlight the significant risks associated with ocular tattooing, underscoring that the procedure is not devoid of serious consequences. 

Case 1 involves a patient who presented with severe pain and inflammation shortly after the procedure. Upon examination, the patient exhibited conjunctival hyperemia (extensive redness) and corneal epitheliopathy, signs of irritation, and damage to the eye’s outermost layer. The inflammatory response raised urgent concerns about the long-term effects on ocular health, suggesting that the tattooing process could provoke a substantial tissue reaction.

Case 2 describes another patient who similarly experienced acute discomfort and inflammation after the procedure. This individual’s condition was more severe, as medical assessment revealed signs of retinal detachment. The immediate need for intervention in this case highlights the potential for catastrophic outcomes associated with cosmetic eyedrop procedures.

The authors emphasize the critical need for practitioners to exercise caution when considering eyeball tattooing. Given that the immediate effects can range from mild discomfort to severe complications such as retinal detachment, thorough patient selection, proper informed consent, and careful pre-operative assessment are essential to minimize risks. They argue for increased awareness among both patients and healthcare providers about the dangers inherent to such cosmetic interventions (Level IV).

Shalini et al. [16] investigate a serious incident involving an individual who attempted to perform eyeball tattooing on themselves. This self-intervention led to unintended globe penetration, illustrating the severe risks associated with untrained attempts at cosmetic eye procedures. The case describes a young patient who sought to enhance their appearance by tattooing the sclera. However, due to a lack of professional guidance and the inherent challenges of performing such a sensitive procedure independently, the outcome was disastrous. During the self-tattooing attempt, the patient inadvertently punctured the globe, causing substantial damage to the ocular structure.

Upon presentation to a medical facility, the patient exhibited prominent symptoms indicative of globe penetration, including severe pain and loss of vision. A thorough ocular examination confirmed the severity of the injury, necessitating urgent medical intervention. The report details the subsequent surgical procedures undertaken to address the globe penetration, which involved repairing the damage and attempting to restore the integrity of the eye. This case underscores several critical issues surrounding the trend in cosmetic eyeball tattooing. First, it highlights the dangerous consequences of individuals attempting medical procedures without the requisite expertise, particularly in delicate areas such as the eyes. Self-attempted procedures pose a high risk of complications and can lead to irreversible damage, as evidenced by this case (Level IV).

Jalil et al. [17] present a significant case involving an individual who experienced severe complications following an attempted eyeball tattooing procedure. This case contributes to the growing body of evidence concerning the risks associated with the cosmetic practice of eyeball tattooing. The report describes a patient who pursued eyeball tattooing to enhance the cosmetic appearance of their sclera. Despite the procedure’s rising popularity, especially among those seeking unique body art, it poses substantial risks, particularly when conducted outside a professional medical setting.

In this case, after undergoing the tattooing procedure, the patient presented with symptoms indicative of ocular trauma, including pain and visual disturbances. Upon examination, the authors identified both globe penetration and the deposition of tattoo pigment within the intraocular space. This was a particularly rare and alarming complication, as it demonstrated not only the possibility of physical damage to the eye but also the movement of foreign pigment into critical ocular structures.

The investigation included imaging studies to assess the extent of the injury and determine the appropriate course of treatment. The authors discussed the types of complications that can arise from eyeball tattooing, including inflammatory responses, infection, and long-term effects on vision (Level IV).

Ostheimer et al. [18] investigate a rare but significant complication that may arise from the practice of tattooing. The authors document several cases of uveitis, an inflammation of the uveal tract within the eye, that were linked to tattoo procedures, thereby highlighting a potential adverse effect of this increasingly popular cultural phenomenon. In the report, the researchers present clinical data on patients who developed uveitis following various types of tattooing, including skin tattoos and ocular tattoos. They detail the symptoms experienced by these patients, such as blurred vision, photophobia, and ocular discomfort, which prompted them to seek ophthalmic evaluation. 

The authors conducted a thorough examination, emphasizing the importance of considering a patient’s history of tattooing when diagnosing unexplained uveitis. Through a series of case studies, they noted that the onset of uveitis in these patients usually occurred within a short time frame after receiving tattoos, indicating a possible direct correlation. Furthermore, the study discusses the histopathological findings and potential mechanisms behind tattoo-related uveitis, such as the body’s inflammatory response to ink pigments or other materials introduced into the skin.

The authors also explore the implications for management and treatment of patients suffering from tattoo-associated uveitis. They stress the significance of early detection and appropriate therapeutic interventions, including corticosteroids, to mitigate the inflammatory response and preserve vision. Additionally, the authors call for greater awareness among practitioners regarding this potential risk and encourage discussions about the possibility of uveitis with patients who choose to become tattooed (Level IV).

Giulbudagian et al. [19] investigate the regulatory landscape surrounding tattoos and permanent makeup, with a particular focus on safety concerns related to materials used in these procedures. While the article primarily addresses the broader implications of tattoos and permanent makeup, it also touches on the adverse effects associated with ocular tattoos, highlighting the unique risks involved in ocular applications. The article examines the general safety profile of inks and pigments used for tattoos, including those applied to the ocular region. The authors note that ocular tattoos, which can involve the injection of pigments into the sclera, pose additional risks due to the delicate nature of eye anatomy. Adverse effects from ocular tattooing can include inflammation, infection, and severe complications such as vision loss. The authors emphasize that pigments used for body tattoos may not have been adequately tested for safety within the eye, which raises concerns about long-term biocompatibility and potential toxicity.

The authors also critique the lack of comprehensive regulation and oversight concerning the materials used for cosmetic purposes, including ocular tattoos. They stress that many inks contain hazardous substances, such as heavy metals and other chemicals that could provoke allergic reactions or inflammatory responses. The risks are further compounded by the procedural techniques employed, which vary significantly among practitioners, many of whom may not be adequately trained in the ocular environment (Level IV).

Wang et al. [20] discuss two clinical cases involving complications from episcleral tattooing, a cosmetic procedure where pigment is injected into the episcleral tissue. The authors provide insights into the adverse effects experienced by the patients and the subsequent clinical management required in an acute ophthalmic setting. In their report, the authors describe two patients who presented to the acute ophthalmic clinic after undergoing episcleral tattooing. Both patients reported experiencing ocular symptoms, including discomfort, redness, and visual disturbances, following the procedure. Upon examination, clinicians found signs of inflammation and irritation at the site of the tattoo, along with other complications potentially associated with the pigment used.

The cases highlighted various complications, including granulomatous reactions and potential risk of intraocular inflammation. The authors emphasize the need for careful patient evaluation and monitoring following episcleral tattooing, as well as the importance of thorough pre-procedure counseling regarding the risks involved. They discuss how these complications necessitate prompt treatment and may lead to esthetic dissatisfaction, undermining the intended purpose of the procedure (Level IV).

Tubek et al. [1] examine the implications of ocular tattoos through the case of a young female patient who underwent this increasingly popular cosmetic procedure. The authors highlight both the esthetic motivations behind the procedure and the significant ocular complications that may arise, providing a crucial understanding for ophthalmologists confronted with similar cases. The case report details the patient’s experience following her decision to tattoo her sclera with a bright color. Initially, the result met her esthetic expectations. However, she soon presented with adverse ocular effects, including inflammation and discomfort, necessitating intensive ophthalmological care. The article emphasizes that such complications are not uncommon and can range from mild irritation to serious issues like inflammation, scarring, and potential vision loss.

The authors conduct a comprehensive review of existing literature on ocular tattoos, finding a limited number of studies addressing the safety and outcomes of this practice. They note that many individuals pursuing ocular tattooing are often unaware of the risks involved, which may include allergic reactions to pigments, infections, and long-term changes to ocular health. The materials used in the procedure, such as inks developed for body tattoos, are not always suitable for use in the eye due to their chemical composition, generating concerns about biocompatibility.

The review underscores the need for increased awareness among both practitioners and patients regarding the associated risks of ocular tattoos. The authors call for stricter regulations in the field of cosmetic eye enhancements to protect patients’ health and ensure that practitioners are well-informed about the potential complications involved (Level IV).

### 3.2. Conjunctival Tattoo Adverse Effects

Dixon et al. [21] detail a serious incident of inadvertent intravitreous ink injection during a subconjunctival tattooing procedure. This unfortunate event resulted in intraocular inflammation and retinal trauma, highlighting the risks involved when such procedures are performed.

During the subconjunctival tattooing procedure, which is often sought after for cosmetic purposes, tattoo ink was mistakenly injected into the vitreous cavity of the eye. This led to the patient’s presentation of ocular inflammation, characterized by redness, discomfort, and deterioration of visual function. The presence of the foreign material within the eye prompted a toxic reaction, resulting in significant retinal damage. The case underscores the critical need for practitioners to adhere to precise injection techniques and to understand the potential consequences of injecting materials close to sensitive ocular structures.

Complementing this case report, the literature review regarding eyelid cosmetic enhancements provides a broader perspective on the range of ocular adverse effects associated with various cosmetic procedures. This review highlights that enhancements such as permanent eyeliner and tattoos can lead to complications including dry eye symptoms, allergic reactions to cosmetic materials, and complications arising from improper placement or technique. The proximity of these procedures to the eye increases the risk of adverse events, with some patients experiencing chronic ocular surface disorders and impairments in tear film stability due to meibomian gland dysfunction (Level IV).

Rodríguez-Avila et al. [22] address severe complications arising from conjunctival tattooing, specifically focusing on a case where inadvertent penetration of the ocular globe occurred. This scenario sheds light on the potential risks associated with cosmetic procedures that involve the delicate structures of the eye. In this report, the authors describe a patient who underwent a conjunctival tattooing procedure aimed at esthetic enhancement. However, during the procedure, the tattooing technique inadvertently resulted in penetration of the ocular globe, leading to the introduction of foreign material into the vitreous cavity. The authors highlight the clinical manifestations of this complication, which included symptoms of pain, inflammation, and compromised vision.

The study employs a combination of clinico-pathological correlation and advanced imaging techniques, including scanning electron microscopy and X-ray microanalysis, to evaluate the effects of the tattoo ink on ocular tissues. The pathological examination revealed significant intraocular inflammation, and the presence of foreign material in the vitreous confirmed the serious nature of the complication. Microscopic analysis further elucidated the morphological changes within the ocular tissues, providing insights into the toxicological effects of the injected ink.

The authors emphasize that the introduction of ink into the eye can provoke severe inflammatory responses and may lead to complications such as retinal detachment, intraocular hemorrhage, or permanent vision loss. This case underlines the importance of understanding the potential adverse effects of cosmetic procedures, particularly those involving injections near or within ocular structures (Level IV).

Da Cruz et al. [5] present a significant clinical case focusing on the complications arising from conjunctival tattooing, a procedure that involves applying cosmetic dye to the conjunctiva. The authors detail a case where inadvertent penetration of the globe occurred during the tattooing process, leading to serious ocular complications.

The case involves a patient who sought conjunctival tattooing for esthetic reasons. Unfortunately, during the procedure, the tattooing tool accidentally penetrated the globe, causing an array of complications. Following the incident, the patient presented with symptoms including pain, redness, and vision changes. Clinical examination revealed not only the unintended penetration but also signs of infection and potential damage to intraocular structures. The authors highlight the importance of immediate medical intervention to address such injuries, underscoring the necessity for accurate and effective management to prevent long-term visual impairment.

The article emphasizes that conjunctival tattooing is often perceived as a relatively simple procedure; however, it carries significant risks, particularly if performed by practitioners lacking specialized training in ocular anatomy and safe techniques. The authors argue for increased awareness and caution among both practitioners and patients regarding the potential for serious complications associated with cosmetic eye procedures (Level IV).

Cruz et al. [6] discuss the rising trend in conjunctival tattooing as a form of body modification and highlight the associated ocular adverse effects. Conjunctival tattooing involves the injection of pigments into the conjunctiva and is increasingly sought after by individuals wishing to enhance their appearance. 

While motivated by esthetic considerations, conjunctival tattooing poses significant risks that can lead to serious ocular complications. The authors emphasize that improper technique, lack of knowledge of ocular anatomy, and the use of non-sterile instruments can result in adverse effects such as infection, inflammation, and severe damage to the eye structures. One of the most concerning complications is inadvertent globe penetration, which can lead to vision loss. Additionally, the presence of foreign substances in the eye can provoke chronic inflammatory responses and induce other complications, such as scar formation, impaired tear film stability, and changes in intraocular pressure.

The authors argue that the lack of regulations and standardization in the practice of conjunctival tattooing raises concerns about patient safety. Many procedures are conducted outside clinical settings by practitioners who may not have adequate training in ocular anatomy or the necessary skills to manage potential complications. This situation underscores the pressing need for healthcare professionals to effectively educate patients about the risks of conjunctival tattooing, ensuring informed consent is obtained before any procedures are performed (Level IV).

Li et al. [23] report a unique case of an episcleral mass formation as a complication following ocular surface tattooing. The authors explore the implications of this adverse effect, thereby contributing to the understanding of the potential risks associated with this esthetic practice. The case focuses on a patient who underwent ocular surface tattooing, intending to enhance the cosmetic appearance of their eyes. This procedure involves implanting pigment under the conjunctiva. While often perceived as a relatively simple and harmless cosmetic modification, ocular surface tattooing can lead to significant complications. 

Approximately six weeks after the procedure, the patient presented with noticeable discomfort and a visible mass in the episcleral region. Clinical examination revealed a raised, pigmented lesion that had developed as a secondary effect of the tattooing. The authors detail the diagnostic workup, which involved imaging and histopathological examination, confirming that the mass was related to the irritative changes and foreign body response due to the tattoo pigment. The authors discuss that the formation of an episcleral mass serves as a stark reminder of the body’s potential reactions to foreign substances. The phenomenon suggests that the introduction of pigment into the ocular surface can elicit an inflammatory reaction, leading to the development of masses or lesions that may complicate vision and necessitate further medical evaluation and treatment (Level IV).

### 3.3. Corneal Tattoo Adverse Effects

D’Oria et al. [24] investigate the implications of cosmetic keratopigmentation, a procedure that involves the implantation of pigment into the corneal stroma to enhance the cosmetic appearance of the eye in patients with visible corneal opacities. While the esthetic benefits of this procedure are notable, the authors highlight several potential adverse effects that can arise from keratopigmentation, warranting careful consideration from both practitioners and patients. The study reports various ocular adverse effects associated with keratopigmentation. One significant concern is the risk of inflammation, which can arise as the body reacts to the implanted pigment. Patients may experience symptoms such as redness, discomfort, and photophobia, indicating an inflammatory response. Inflammation can lead to complications such as scarring or further opacification, potentially compromising vision.

In addition to inflammation, infection is a risk that the authors identify as a critical concern. Any surgical procedure on the eye carries the potential for introducing pathogens, and keratopigmentation is no exception. Infectious complications can result in serious consequences, including corneal ulcers or endophthalmitis, which can threaten both the integrity of the eye and the patient’s vision.

Another adverse effect noted is pigment migration. Over time, pigment particles may shift from their original site within the corneal tissue. This migration can alter the intended cosmetic outcome, leading to unsatisfactory results and necessitating further intervention or removal of the pigment (Level IIIa).

Ng et al. [25] present a concerning case involving an individual who attempted to perform a self-tattooing procedure on their eyeball, which resulted in a severe injury characterized by corneoscleral perforation. This situation brings to light the risks associated with cosmetic procedures influenced by trends on social media platforms. The authors provide a detailed account of the case, describing how the individual engaged in self-tattooing as a form of personal expression, capitalizing on the growing trend in body modifications showcased on social media. However, the lack of professional oversight and the inherent risks of such an invasive procedure led to significant ocular trauma. The patient presented with symptoms including vision loss, pain, and redness, prompting immediate medical attention.

Upon examination, the medical team discovered corneoscleral perforation, which is a serious condition that can lead to further complications, including endophthalmitis, retinal detachment, and potential loss of the eye. The authors emphasize that such outcomes underline the critical importance of educating the public about the dangers of unregulated body modification practices, particularly those that involve sensitive areas like the eyes. The authors discuss the implications of social media in normalizing and promoting risky behaviors, particularly among younger demographics who may be influenced by viral trends and peer actions. They argue for increased awareness and vigilance regarding the potential consequences of self-tattooing and other similar practices, encouraging discussions around safe body modification and the importance of seeking professional assistance for cosmetic procedures (Level IV).

Paulo et al. [26] presents a unique and alarming case of self-harm involving ocular tattooing. The authors document an incident involving an individual who attempted to modify the color of their eyes by injecting tattoo ink directly into the anterior chamber of the eye. This case illustrates the severe complications that can arise from unsafe and unregulated cosmetic procedures performed by individuals without medical expertise. The patient in this report performed a suboptimal technique of injecting ink, which resulted in immediate adverse effects, including significant ocular inflammation, pain, and vision disturbances. The procedure led to the deposition of tattoo ink within the anterior chamber. Following the injection, the patient experienced a pronounced inflammatory response, which the authors detail, highlighting the detrimental effects of introducing foreign substances into the eye.

To evaluate the complications arising from this incident, the authors conducted a thorough ophthalmological examination, revealing signs of acute anterior chamber inflammation, associated corneal endothelial damage, and increased intraocular pressure. These findings underscore the severity of the situation, as such inflammation could potentially lead to long-term visual impairment or blindness.

The article emphasizes the risks associated with cosmetic procedures that involve the eyes, particularly when performed in non-clinical settings or by individuals lacking appropriate medical training. The authors stress the importance of public awareness regarding the dangers of self-administered cosmetic procedures and the necessity for safe and regulated practices in cosmetic eye enhancements (Level IV).

### 3.4. Eyelid Tattoo Adverse Effects

Yan et al. [27] examine the risks of ocular injuries associated with various commercial cosmetic procedures. The authors review specific incidents and case reports where clients suffered ocular injuries during cosmetic treatments, including procedures such as eyelash extensions, eyebrow tattooing, and the application of injectable fillers. The injuries reported range from chemical burns and allergic reactions to more severe complications like corneal abrasion and vision impairment.

The authors analyze the mechanisms behind these injuries, emphasizing factors such as the improper use of products, inadequate training of practitioners, and lack of adherence to safety precautions. They discuss the implications for both practitioners and patients, underscoring the necessity of proper education and training in cosmetic procedures to prevent such complications. The article also highlights the importance of informed consent, where practitioners should effectively communicate potential risks to clients. The authors advocate for the implementation of safety protocols across the industry to minimize the risk of ocular injuries and improve overall patient safety during cosmetic procedures (Level IV).

Bee et al. [28] present a clinical case highlighting a rare but significant occurrence of tattoo granuloma located on the eyelid. The article outlines the challenges in diagnosing such lesions, as they can easily be confused with more serious conditions like carcinoma. The authors detail the case of a patient who developed localized swelling and pigmentation on the eyelid following the application of a cosmetic tattoo. Initial assessments misled the healthcare team into suspecting the presence of an eyelid carcinoma due to the atypical presentation, which included the granulomatous reaction. The differential diagnosis drew attention to the need for thorough examination and consideration of a history of tattooing, which was pivotal in redirecting the diagnostic focus.

Histopathological analysis revealed that the lesion consisted of a dense infiltrate of histiocytes, multinucleated giant cells, and lymphocytes, consistent with a granulomatous response to foreign material, specifically the pigments used in tattooing. This finding was critical in distinguishing the tattoo granuloma from malignancies and observing its benign nature.

The authors emphasize the importance of recognizing tattoo-related complications, including granulomas, as cosmetic tattoos become increasingly popular. They advocate for awareness among ophthalmology practitioners and the general public regarding potential adverse effects that may arise post-tattoo, especially in sensitive areas like the eyelids (Level IV).

Lee et al. [29] examine the ocular complications associated with eyelid tattooing, a cosmetic procedure that is gaining popularity for its ability to enhance appearance by creating a permanent eyeliner effect. While this practice is often considered safe, the study highlights significant adverse effects on ocular health, specifically concerning the meibomian glands and tear film stability. The research included a cohort of patients who underwent eyelid tattooing, with detailed ophthalmic evaluations conducted before and after the procedure. The primary focus was on assessing meibomian gland function, as these glands are integral to producing the lipids that stabilize the tear film and prevent evaporation. A key measurement in the study was tear break-up time, which assesses the stability of the tear film.

The results indicated a notable loss of meibomian gland density following eyelid tattooing, accompanied by decreased tear break-up time. The authors reported that the trauma from the tattooing process, which involves the insertion of ink into delicate eyelid skin, may disrupt the structure and function of the meibomian glands, leading to insufficient lipid production. This dysfunction can contribute to symptoms associated with dry eye disease, including discomfort, visual fluctuations, and ocular irritation.

The findings underscore the clinical significance of understanding the potential ocular consequences of cosmetic procedures such as eyelid tattooing. While the esthetic benefits may appeal to many, this study serves as a critical reminder of the accompanying risks, urging cosmetic practitioners and patients to engage in informed discussions about potential eye-related complications pre- and post-procedure (Level IV).

Van der Bent et al. [30] present a comprehensive review of complications associated with tattoos and permanent makeup. The authors analyzed 308 cases of tattoo-related complications, aiming to provide a detailed understanding of the various adverse reactions and their frequencies. The study revealed that the most common complications were allergic reactions (24.7%), followed by granulomas (14.3%), and infections (12.3%). The authors also noted that skin reactions, such as contact dermatitis, erythema, and pruritus, were common occurrences. Other complications included scarring, keloid formation, and damage to surrounding skin tissue.

The analysis showed that the majority of complications (71.5%) occurred within the first six months after tattooing or permanent makeup application. The authors emphasized the importance of proper aftercare, client education, and a thorough understanding of the risks involved in these procedures (Level IIIb).

De et al. [31] discuss a serious complication stemming from cosmetic procedures involving the injection of pigment into the eyelids, commonly referred to as blepharopigmentation. This technique, which is intended to enhance the appearance of the eyelids, can lead to various adverse effects if not performed with precision and caution. The authors present a case study of a patient who experienced a full-thickness eyelid penetration during the procedure. This incident resulted in significant trauma to the eyelid and underlying structures, emphasizing the potential risks associated with improperly administered cosmetic eyelid treatments. The paper outlines the immediate consequences of the injury, which included pain, swelling, and potential complications such as infection or impaired vision.

The authors highlight that the cosmetic practice of blepharopigmentation, while popular for its esthetic benefits, carries inherent risks, particularly when performed by inadequately trained practitioners. The authors express concern over the lack of standardized training and regulation in the field of cosmetic dermatology, which can lead to dangerous outcomes. They stress the importance of using appropriate techniques and tools to prevent complications during such procedures (Level IV).

Lu et al. [32] examine a significant complication associated with the cosmetic procedure of applying permanent eyeliner. The authors detail the experience of a patient who developed bilateral diffuse lamellar keratitis following the treatment. The report outlines that the patient underwent the permanent eyeliner tattoo procedure, which involves implanting pigment into the eyelid skin. Shortly after the procedure, the patient experienced symptoms including blurred vision, discomfort, and irritation. Upon further ophthalmic examination, the presence of diffuse lamellar keratitis was diagnosed, indicating a severe inflammatory reaction that can have lasting effects on vision if not properly addressed.

The authors emphasize that while cosmetic interventions like permanent eyeliner are widely sought for their esthetic benefits, they are not without risks. The authors point out that diffuse lamellar keratitis, particularly in the context of eyelid tattooing, is a rare but serious complication that might arise due to several factors, including the implantation technique, the materials used, or the body’s immune response to foreign substances in the eye region.

The article highlights the importance of proper technique and selection of materials in preventing such adverse reactions. The authors recommend caution and thorough pre-procedure consultation to inform patients about potential risks and encourage them to seek treatment from qualified professionals. They also suggest that practitioners should be aware of the signs of DLK and other complications arising from cosmetic procedures to facilitate timely intervention (Level IV).

Goldberg et al. [33] describe a case where a patient developed corneal pigmentation as an unintended consequence of a cosmetic procedure known as blepharopigmentation. The authors detail the case of a patient who underwent blepharopigmentation and subsequently presented with symptoms including visual disturbances and discomfort. Upon examination, it was discovered that the pigment had inadvertently migrated into the cornea, leading to corneal pigmentation. This condition can result in various complications, including potential vision impairment, depending on the extent and location of the pigmentation.

The authors highlight several critical factors that could contribute to this adverse outcome, including the skill level of the practitioner, the technique used during the procedure, and the types of pigments employed. They emphasize that proper techniques and precautions are essential to minimize the risk of pigment migration into the eye. The authors stress the importance of educating both practitioners and patients about the potential risks associated with cosmetic eye procedures. They advise that practitioners must be trained adequately and keep up-to-date with best practices to avoid complications such as inadvertent corneal pigmentation.

Furthermore, the report serves as a reminder of the need for careful patient screening and informed consent, ensuring that patients are fully aware of the risks and benefits connected with procedures like blepharopigmentation. The authors also suggest that the rise in popularity of such cosmetic procedures necessitates increased vigilance and standardization among practitioners to ensure patient safety (Level IV).

Gulmez Sevim et al. [34] investigate the complications that can arise following the cosmetic procedure of blepharopigmentation, focusing specifically on the occurrence of severe corneal abrasions. The authors present a case involving a patient who underwent blepharopigmentation. Shortly after the procedure, the patient reported experiencing discomfort and visual disturbances. Upon examination, it was found that the patient had developed severe corneal abrasions, a significant ocular complication that can lead to pain, increased risk of infection, and potential long-term vision issues.

The authors explore the various factors that may contribute to the development of corneal abrasions during or after blepharopigmentation. These include improper technique, inadequate post-procedural care, and potential allergic reactions to the pigments used. The authors emphasize that corneal abrasions can occur due to the introduction of foreign substances near the eye, which may compromise the integrity of the corneal epithelium.

The study underscores the importance of employing strict aseptic techniques and ensuring that practitioners are well-trained in the procedure to minimize the risk of complications. They advocate for thorough pre-operative assessments to identify any contraindications and enhance patient safety. Additionally, the authors highlight the necessity of clear post-operative instructions for patients to reduce the risk of abrasions and other complications (Level IV).

Bussel et al. [35] examine the potential ocular complications and risks associated with cosmetic eyelid tattoos. The authors discuss how cosmetic eyelid tattoos involve the application of pigments to the eyelid margins, a practice that can lead to several complications. One of the central themes of the article is the potential for these tattoos to contribute to the development of ocular surface diseases. The procedure can pose risks such as irritation, allergic reactions, and infections, which may compromise the health of the ocular surface.

The authors highlight that the pigments used in cosmetic tattoos may induce inflammation or trigger sensitivities, leading to conditions such as conjunctivitis or dry eye syndrome. Additionally, they point out that unintended migration of pigment onto the ocular surface may occur, potentially causing further complications. The article emphasizes the importance of proper technique and hygiene during the application of cosmetic eyeliner tattoos, along with the necessity of using high-quality materials. Moreover, the authors recommend that practitioners must be aware of the risks involved in such procedures and should provide thorough pre- and post-procedure counseling to patients (Level IV).

Masud et al. [9] explore the ocular complications associated with various cosmetic procedures, particularly focusing on eyelid cosmetics and their potential adverse effects. One of the notable concerns discussed is the introduction of ocular tattoos as part of esthetic enhancements. The research highlights that while ocular tattoos can improve the appearance of the eyelids, they may also pose significant risks to eye health. The article outlines several adverse effects linked to ocular tattoos, primarily due to the use of pigments that may not be biocompatible. Complications can range from temporary irritations to severe conditions affecting vision. Potential adverse effects mentioned in the article include allergic reactions, inflammatory responses, and debilitating conditions such as infections or scarring. Moreover, the permanent nature of tattoos raises concerns over the management of complications, as removal is often complicated and may lead to further ocular damage. 

The authors also emphasize the lack of comprehensive studies investigating the long-term implications of ocular tattoos, leaving a knowledge gap in understanding their safety profile. They point out that the ocular surface anatomy is sensitive, and even minimal disturbances from tattoos can lead to significant ocular morbidity. The article calls for greater regulatory oversight in the application of these cosmetic enhancements to ensure patient safety and raise awareness among practitioners about potential complications (Level V).

Kojima et al. [36] investigate the ocular surface effects that can arise following eyelid tattooing. The authors present findings from a study that examines changes in tear film stability and the health of the ocular surface in patients who have undergone this cosmetic procedure. In their study, the authors evaluated patients who had received eyelid tattoos and compared their tear film and ocular surface conditions to a control group of individuals without eyelid tattoos. Various assessments were conducted, including measurements of tear break-up time, Schirmer’s test scores (to assess tear production), and ocular surface staining to evaluate any damage or abnormalities on the eye surface.

The results indicated that patients with eyelid tattoos exhibited significant abnormalities in tear film stability and ocular surface health compared to the control group. There were noticeable reductions in tear break-up time and Schirmer’s test values, indicating that these individuals may be experiencing dry eye symptoms and ocular surface irritation as a result of the tattooing procedure. The authors hypothesize that the trauma from tattooing and potential exposure to tattoo pigments may contribute to these ocular surface changes (Level IV).

Seol et al. [37] present a clinical case that highlights a rare but significant complication following cosmetic eyelid tattooing. The authors describe the case of a patient who developed meibomian gland dysfunction after undergoing the eyelid tattooing procedure. The patient presented with symptoms including ocular irritation, dryness, and redness, prompting further investigation.

Upon examination, the authors found that the patient had notable signs of meibomian gland dysfunction, which were likely exacerbated by the mechanical trauma and potential inflammatory response associated with the tattooing procedure. The authors carefully documented the diagnostic criteria used to evaluate meibomian gland dysfunction and the associated clinical findings. The article emphasizes the need for greater awareness among practitioners and patients regarding the potential adverse effects of cosmetic eyelid tattooing, particularly concerning the health of the ocular surface and meibomian glands. The authors advocate for thorough pre-procedure assessments and informed consent to ensure that patients are aware of the risks involved (Level 4).

Peters et al. [38] discuss a case of excessive pigment diffusion following a blepharopigmentation procedure. The authors describe the clinical presentation of a patient who experienced significant lateral spread of pigment in the lower eyelid region after undergoing this procedure. The patient presented with an unintended cosmetic outcome that resulted in a darkened appearance beyond the intended tattoo area, leading to esthetic concerns.

The report details the methodology of the blepharopigmentation, the characteristics of the pigment used, and the procedural technique. The authors also review possible factors contributing to the extensive pigment spread, including the application technique, the properties of the tattoo pigment, and the anatomical features of the eyelid. The case highlights the importance of ensuring an appropriate technique and careful handling of the tattooing procedure to minimize the risk of complications such as pigment spread (Level IV).

De Cuyper et al. [39] provide a comprehensive overview of the potential complications associated with cosmetic tattooing procedures, which include techniques such as permanent makeup and body art. The author categorizes complications into several types: immediate reactions, delayed reactions, and long-term issues. Immediate complications may include pain, swelling, and allergic reactions to pigments or anesthetics used during the procedure. Delayed reactions can present as infections, granulomas, or keloids developing at the site of the tattoo, sometimes as a result of improper hygiene practices or allergic responses to ink components.

The author discusses specific pigment-related issues, noting that various pigment formulations can vary in safety and biocompatibility. The risk of systemic allergic reactions to certain pigments is highlighted, along with implications for individuals with pre-existing conditions or skin sensitivities. Long-term complications are also addressed, including the fading of pigments, which may lead to changes in the appearance of the tattoo over time, and complications arising from removing cosmetic tattoos, such as scarring or changes in skin texture (Level IV).

Morrison et al. [40] explore a novel perspective on the potential ocular complications associated with cosmetic tattoos, specifically tattooed eyeliner. The authors address a growing concern among patients who undergo cosmetic tattooing procedures and subsequently experience symptoms of dry eye. The article begins by discussing the increasing popularity of eyelid tattoos as a way to enhance the appearance of the eyes without the daily hassle of applying traditional makeup. However, as more individuals opt for this procedure, reports of adverse effects, such as dry eye symptoms, have also become more common. The authors present a case study of a patient who developed significant dry eye symptoms after receiving eyelid tattooing, raising questions about the connection between the cosmetic procedure and ocular health.

The authors delve into the potential mechanisms that might link eyelid tattooing to dry eye conditions, including irritation caused by the ink, inflammation of the eyelid margins, or disruption of the tear film due to foreign substances. They emphasize the importance of considering each patient’s unique ocular surface condition, history, and the specific materials used in the tattooing process when diagnosing and treating dry eye symptoms arising after such procedures.

The authors propose that optometrists should take a proactive approach in educating patients about the potential risks associated with cosmetic eyelid tattoos. They advocate for a thorough pre-procedure assessment of ocular surface health and post-procedure follow-up to monitor for any complications. The authors suggest developing a treatment protocol for patients who experience dry eye symptoms following tattoo eyeliner procedures, which may include the use of artificial tears, lid hygiene measures, and consultation with other healthcare providers if necessary (Level IV).

Liao et al. [41] present a case report highlighting a rare but significant pigmentation disorder resulting from cosmetic eyeliner tattoos. The authors detail the case of a patient who developed late-onset melanopenic hypomelanosis at the site of a cosmetic eyeliner tattoo. Initially, the patient had undergone the procedure without immediate complications. However, several years later, she noticed a progressive loss of pigmentation around the tattooed area. Upon examination, the authors discovered that the eyelid region exhibited a distinct and irregular pattern of hypopigmentation, contrasting with the surrounding skin. This unusual finding suggested that the tattoo pigment may have triggered a localized inflammatory response leading to a reduction in melanin production in the affected area.

The authors conducted a thorough review of the literature and noted that while complications from tattoos and permanent makeup have been documented, the specific presentation of melanopenic hypomelanosis following cosmetic tattooing is rare. They discuss potential mechanisms for this pigmentation loss, including inflammatory responses, alteration of melanocyte function, and the role of tattoo ink itself.

The study underscores the necessity for dermatologists and practitioners performing cosmetic tattooing to be aware of potential delayed complications. The authors emphasize that patients should be informed about these risks before undergoing such procedures. They also call for further research to better understand the relationship between cosmetic tattoos and skin changes, especially in relation to pigmentation (Level IV).

Vagefi et al. [42] discuss the complications and adverse reactions associated with permanent eyelid tattoos. The authors present a series of cases from their clinical experience, highlighting the types and frequencies of various reactions that patients may encounter following the procedure. The study reports on the experiences of several patients who underwent the application of permanent eyelid tattoos and subsequently developed a range of adverse effects. Common complications noted include allergic reactions, infections, and granulomatous responses. The authors also documented cases of pigment migration and changes in the appearance of the tattoo over time, which could lead to patient dissatisfaction.

The article emphasizes the importance of informed consent, where patients should be made aware of potential risks and adverse effects associated with the procedure. The authors recommend thorough pre-procedure evaluations and post-procedure follow-ups to mitigate risks and address any complications that arise effectively (Level IV).

Yoon et al. [43] investigate the potential impact of cosmetic eyeliner tattooing on the health of the ocular surface and the function of meibomian glands. With the rising popularity of semi-permanent makeup, particularly eyelid tattoos, the study examines the relationship between eyeliner tattoos and meibomian gland dysfunction, which can lead to dry eye symptoms, discomfort, and visual impairment. Given that eyeliner tattoos involve the application of pigments near the eye, the researchers aimed to determine if the procedure influences the health of the ocular surface and the function of the meibomian glands.

To assess this, the authors conducted a clinical evaluation of patients who had received eyeliner tattoos. They measured various parameters related to ocular surface health, including tear break-up time, ocular surface staining, and meibomian gland function. Comparisons were made between patients with eyeliner tattoos and a control group without such cosmetic enhancements. The findings indicated that patients with eyeliner tattoos exhibited a higher prevalence of meibomian gland dysfunction compared to the control group. Specifically, several parameters indicative of compromised ocular surface health, such as decreased tear break-up time and increased ocular surface staining scores, were seen in the tattooed group. The study concludes that eyeliner tattooing may compromise the health of the meibomian glands and the overall ocular surface, leading to symptoms associated with dry eye disease (Level IV).

### 3.5. Eyebrow Tattoo Adverse Effects

Lee et al. [44] discuss two clinical cases involving complications related to eyelash and eyebrow tattooing procedures, specifically focusing on the phenomenon of pigment fanning. In this report, the authors describe two patients who experienced pigment fanning, a cosmetic issue characterized by the unintended spreading or diffusion of pigment from the original tattooed area. This complication resulted in an undesirable esthetic appearance, affecting the patients’ satisfaction and prompting them to seek corrective measures.

The article outlines the procedures undergone by the patients, including the techniques and materials used for the tattooing process. It also reviews potential causes of pigment fanning, such as the specific ink formulations, the needle technique, and post-procedure factors that may contribute to this complication. The authors emphasize the importance of choosing qualified professionals for cosmetic tattooing procedures and the need for thorough patient education regarding potential risks and complications. They recommend careful consideration of techniques to minimize the risk of pigment fanning and encourage monitoring and appropriate management of complications when they occur (Level IV).

Gaspari et al. [45] examine a case involving a patient who developed significant ocular symptoms attributed to recent permanent eyebrow tattooing. The study highlights the delayed onset of papillary conjunctivitis as a rare complication of this cosmetic procedure and emphasizes the need for awareness of potential long-term effects of tattooing practices on ocular health.

The case report details the patient’s clinical presentation, which included redness, itching, and discomfort in the eyes that developed several months after the eyebrow tattooing procedure. Upon examination, the authors noted the presence of papillary conjunctivitis, characterized by chemosis (swelling of the conjunctiva) and prominent papillae on the conjunctival surface. This condition was attributed to an allergic or irritant reaction to the tattoo ink or other materials used during the procedure.

The authors discuss potential contributing factors to the development of conjunctivitis in this patient, including the specific formulation of the tattoo ink, the technique used during application, and the patient’s individual sensitivity to foreign substances. They also explore the possibility of the tattoo ink migrating to the conjunctival area, leading to the inflammatory reaction observed. The authors outline the management strategy for the patient, which included the use of topical antihistamines and corticosteroids to address inflammation and provide symptomatic relief. They also stress the importance of patient education regarding the potential risks associated with cosmetic tattooing, particularly for individuals with sensitive skin or a history of allergic reactions (Level IV).

## 4. Discussion

Tattoos, once considered a purely esthetic personal expression, have gained vast popularity across various demographics. The expanding trend in ocular and periocular tattoos, where ink is applied directly to the conjunctiva or the skin surrounding the eyes, has prompted an increase in the occurrence of associated complications. As practitioners in the medical field, we must critically evaluate the potential risks and complications arising from these procedures to provide the best patient care and inform public knowledge. 

The prevalence of ocular tattoos is difficult to determine, but anecdotal evidence suggests a growing interest in such permanent cosmetic procedures, which now extend to a range of techniques from traditional needle tattoos to more modern innovations like the use of dermal implants. Complications can arise from any of these approaches, potentially leading to significant eye health problems and esthetic concerns. Periocular tattooing, while generally considered less invasive, also poses unique risks that must be thoroughly understood and communicated to patients prior to engagement in such practices. 

In assessing the causes of complications from ocular and periocular tattoos, it is crucial to differentiate between those resulting from the chemical properties of the ink and those caused by mechanical trauma from the tattoo applicator. Chemical complications are often linked to the composition of the ink, which may contain potentially harmful substances such as heavy metals or other allergens that can provoke inflammatory responses, infections, or allergic reactions. Mechanical complications, on the other hand, are primarily associated with the physical process of tattooing, which involves needle penetration and can lead to tissue trauma, improper ink placement, or unintentional injuries to adjacent ocular structures. By clarifying these distinctions, our review provides a nuanced understanding of the etiological factors contributing to the complications of ocular and periocular tattooing, emphasizing the importance of both material safety and procedural precision in mitigating risks (Table 2).

### 4.1. Complications and Their Mechanisms

The complications associated with ocular and periocular tattoos can be categorized into immediate and delayed reactions. 

#### 4.1.1. Immediate Reactions

Immediate complications include bleeding, infection, and allergic reactions. The delicate nature of ocular tissues means that even minor punctures can lead to significant bleeding or hematoma, especially given the vascular supply to the periocular region. Infection can arise due to non-sterile conditions during application, where contaminated needles or inks introduce pathogenic organisms, leading to conjunctivitis or other serious infections such as endophthalmitis [1,3,5,9,19,20,21,26,30,35,39,42]. Hypersensitivity to tattoo pigments is another immediate complication. Reports of allergic reactions to commonly used pigments have been documented. These reactions can manifest as localized inflammation, itching, and discomfort, further complicating the healing process and increasing the risk of secondary infections [9,19,30,35,39,42].

#### 4.1.2. Delayed Reactions

Delayed complications such as granuloma formation and even scarring are also significant concerns. Granulomas may develop as a reaction to foreign substances, in this case, tattoo ink, which stimulates an inflammatory response. This chronic inflammation can lead to unsightly lesions, affecting both visual function and esthetic outcomes [4,9,14,20,23,28,30,35,39,42]. 

#### 4.1.3. Visual Impairment and Eye Injury

One of the most serious long-term complications of ocular tattoos is the potential for significant visual impairment. Inappropriate tattoo placements can lead to damage to the cornea or retina, contributing to vision loss. There have been documented cases where patients experienced acute vision deterioration shortly following the procedure, underscoring the critical need for thorough patient education regarding the risks [5,16,17,22,25].

#### 4.1.4. Other Rare Complications

For scleral tattooing, there are reports of granulomatous anterior uveitis [14], retinal detachment [15]. For conjunctival tattooing, there are reports of foreign material in the vitreous cavity [22] and episcleral mass (due to reaction to a foreign substance) [23]. For corneal tattooing, there are reports of corneoscleral perforation [25] and deposition of tattoo ink within the anterior chamber [26]. For eyelid tattooing, there are reports of tattoo granuloma mimicking carcinoma [28], meibomian gland dysfunction [29,37,43], full-thickness eyelid penetration [31], bilateral diffuse lamella keratitis [32], inadvertent corneal pigmentation [33], tear film instability [29,36], pigment spread [38], dry eye [40], and late-onset melanopenic hypomelanosis [41]. For eyebrow tattooing, there are reports of pigment spread [44] and papillary conjunctivitis [45].

### 4.2. Importance of Practitioner Experience and Patient Education

The rise in ocular and periocular tattoo procedures, often performed outside traditional medical settings, raises concerns about the qualifications of those providing these services. The potential for complications is markedly higher when these procedures are performed by practitioners without adequate training in ocular anatomy and associated risks. The involvement of qualified medical professionals capable of conducting proper pre-operative assessments can reduce complication rates significantly [46,47].

Furthermore, a comprehensive patient education program is essential. This should include detailed discussions about the potential risks and complications associated with tattooing, informed consent practices, and the importance of following post-procedure care instructions rigorously. 

Patients should be made aware of the symptoms that require immediate medical attention, such as excessive redness, swelling, or discharge, which could signal infection or other complications [48].

#### 4.2.1. Regulatory Measures and Legal Implications

Given the nature of complications arising from ocular and periocular tattoos, regulatory measures must be reinforced. Current regulations surrounding tattooing practices vary widely across different jurisdictions, and many areas lack stringent guidelines specifically addressing ocular tattooing. It is crucial for professional medical organizations to devise clear guidelines that govern the practice of ocular tattooing, potentially including certification processes for practitioners.

Legal implications must be considered as well, particularly as litigation related to malpractice in cosmetic procedures becomes increasingly common. Practitioners must not only ensure they obtain informed consent but also maintain clear documentation of patient interactions, care protocols, and any adverse outcomes. This thorough documentation can serve as a protective measure against potential legal repercussions [49,50].

#### 4.2.2. Future Research Directions

In light of the rising trend and accompanying complications, further research into ocular and periocular tattoos is necessary to establish a robust evidence base. Longitudinal studies examining the long-term effects of these tattoos on vision and ocular health are warranted. Investigating the specific inks and pigments associated with reduced risks of complications would also enhance our understanding and improve safety protocols.

Additionally, qualitative studies exploring patient motivations behind choosing ocular tattoos can provide deeper insights into the psychological factors at play, potentially guiding better patient education and care strategies.

## 5. Conclusions

In conclusion, ocular and periocular tattoos pose a unique set of challenges and potential complications that require close examination. As the popularity of these procedures continues to grow, it becomes imperative for healthcare providers to remain vigilant regarding the associated risks. Highlighting the need for informed patient choices, thorough practitioner training, and appropriate regulatory measures will be critical in preventing complications. A collaborative approach that encompasses medical professionals, regulatory bodies, and patients alike can help navigate the complexities of this evolving field while prioritizing patient safety and well-being. 

By fostering an ongoing dialog about the implications of ocular and periocular tattoos, we can better equip our medical community to address the complications inherent in these procedures and promote responsible practice standards that prioritize patient health above esthetic desires.

## Figures and Tables

**Table 1:** This table provides a comprehensive summary of the studies reviewed, highlighting their focus and publication years.

Reference	Authors	Study Focus	Publication Year
[1]	Tubek et al.	Case report on scleral tattoo complications	2019
[2]	Brodie et al.	Episcleral tattoo complications	2015
[3]	Rohl et al.	Pen ink scleral tattoo complications	2021
[4]	Carlisle et al.	Scleral tattoo-induced sarcoid reaction	2023
[5]	da Cruz et al.	Complications of conjunctival tattooing with globe penetration	2017
[6]	Cruz et al.	Overview of conjunctival tattooing adverse effects	2017
[7]	Pitz et al.	Corneal tattooing for cosmetic and therapeutic purposes	2002
[8]	Doganay et al.	Corneal tattooing for esthetic enhancement	2020
[9]	Masud et al.	Eyelid tattoos and associated ocular adverse effects	2019
[10]	Lund et al.	Complications of eyelid tattooing	1986
[12]	Nie et al.	Eyebrow tattoo-associated sarcoidosis	2022
[13]	Ro et al.	Granulomatous tissue reaction from eyebrow tattooing	1991
[11]	Oxford Center for Evidence-Based Medicine	Levels of evidence framework	2009
[14]	Haq et al.	Granulomatous anterior uveitis from scleral tattoo	2021
[15]	Duarte et al.	Short-term complications of eyeball tattoos	2017
[16]	Shalini et al.	Globe penetration due to self-attempted eyeball tattooing	2022
[17]	Jalil et al.	Intraocular tattoo pigment deposition from eyeball tattoos	2015
[18]	Ostheimer et al.	Tattoo-associated uveitis	2014
[19]	Giulbudagian et al.	Safety of tattoos and permanent makeup: regulatory view	2020
[20]	Wang et al.	Episcleral tattooing complications	2020
[21]	Dixon et al.	Intravitreous ink injection complications from tattooing	2018
[22]	Rodríguez-Avila et al.	Globe penetration in conjunctival tattooing	2020
[23]	Li et al.	Episcleral mass from ocular surface tattooing	2020
[24]	D’Oria et al.	Complications of corneal keratopigmentation	2021
[25]	Ng et al.	Corneoscleral perforation from self-tattooing	2019
[26]	Paulo et al.	Anterior chamber tattoo complications	2018
[27]	Yan et al.	Ocular injuries from commercial cosmetic procedures	2020
[28]	Bee et al.	Tattoo granuloma of the eyelid	2014
[29]	Lee et al.	Eyelid tattooing and meibomian gland dysfunction	2015
[30]	Van der Bent et al.	Overview of complications from tattoos and permanent makeup	2021
[31]	De et al.	Full-thickness eyelid penetration during blepharopigmentation	2008
[32]	Lu et al.	Lamellar keratitis from permanent eyeliner tattoo	2017
[33]	Goldberg et al.	Corneal pigmentation following blepharopigmentation	2018
[34]	Gulmez Sevim et al.	Corneal abrasions from blepharopigmentation	2019
[35]	Bussel et al.	Cosmetic eyeliner tattoos and ocular surface disease	2018
[36]	Kojima et al.	Tear film abnormalities after eyelid tattooing	2005
[37]	Seol et al.	Meibomian gland dysfunction after eyelid tattooing	2013
[38]	Peters et al.	Pigment spread after blepharopigmentation	1999
[39]	De Cuyper	Complications of cosmetic tattoos	2015
[40]	Morrison et al.	Dry eye symptoms after eyeliner tattoo	2016
[41]	Liao et al.	Pigmentation disorder from eyeliner tattoo	2013
[42]	Vagefi et al.	Adverse reactions to permanent eyeliner tattoo	2006
[43]	Yoon et al.	Eyeliner tattoo impact on meibomian gland function	2020
[44]	Lee et al.	Pigment fanning in eyelash and eyebrow tattoos	2001
[45]	Gaspari et al.	Papillary conjunctivitis after eyebrow tattooing	2022

**Table 2:** Comprehensive summary of the complications for different types of ocular and periocular tattoos.

Tattoo Type	Complications	Causes	References
Scleral Tattoos	Granulomatous anterior uveitis, retinal detachment, infection, allergic reactions, intraocular inflammation, visual impairment	Chemical reactions to ink, mechanical trauma from applicator	[4,14,15,16,17,18,19,20]
Conjunctival Tattoos	Foreign material in vitreous cavity, episcleral mass formation, infection, intraocular hemorrhage, vision loss, chronic inflammation	Chemical reactions to ink, mechanical injury from needle	[5,6,21,22,23]
Corneal Tattoos	Corneoscleral perforation, pigment migration, inflammation, infection, corneal scarring, long-term vision impairment	Ink-related inflammation, physical trauma from tattooing	[7,8,24,25,26]
Eyelid Tattoos	Meibomian gland dysfunction, tear film instability, dry eye, corneal pigmentation, diffuse lamellar keratitis, granuloma formation, eyelid trauma	Ink toxicity, mechanical impact on eyelid structure	[9,28,29,30,31,32,33,34,35,36,37]
Eyebrow Tattoos	Pigment spread, papillary conjunctivitis, allergic reactions, granulomas, chronic inflammation, esthetic dissatisfaction	Chemical irritants in ink, mechanical disruption of tissues	[12,13,44,45]
